# Voices from Within: Saudi Arabian Women’s Lived Experiences of First-Episode Psychosis, Hospitalisation, and Recovery Pathways

**DOI:** 10.3390/healthcare14131970

**Published:** 2026-07-02

**Authors:** Asrar S. Almutairi, Alya Alghamdi, Norah M. Alyahya, Bader M. Almutairy, Abdulaziz M. Alodhailah, Ashwaq A. Almutairi, Faihan F. Alshaibany, Waleed M. Alshehri, Thurayya Eid

**Affiliations:** 1 Community and Psychiatric Mental Health Nursing Department, College of Nursing, Princess Nourah Bint Abdulrahman University, Riyadh 11671, Saudi Arabia; 2 Community and Psychiatric Mental Health Nursing Department, College of Nursing, King Saud University, Riyadh 11451, Saudi Arabia; 3 Department of Medical-Surgical Nursing, College of Nursing, King Saud University, Riyadh 11451, Saudi Arabia; 4 Monash Nursing and Midwifery, Monash University, Melbourne, VIC 3800, Australia; 5 Department of Nursing Administration and Education, College of Nursing, King Saud University, Riyadh 11451, Saudi Arabia

**Keywords:** psychosis, women, lived experience, recovery, Saudi Arabia, phenomenology, gender-sensitive care, first-episode psychosis, stigma, discharge planning

## Abstract

**Background**: While the consumer experience of psychosis has received significant attention in Western research, a substantial gap exists regarding the experiences of women in the Kingdom of Saudi Arabia (KSA). In this context, religious, cultural, familial, and gender-specific factors uniquely shape the experience of psychosis, help-seeking behaviors, and recovery. This study aimed to explore the lived experiences of Saudi women with psychosis across three phases: first-episode onset, hospitalization or follow-up, and community living after discharge. **Methods**: This hermeneutic phenomenological study, guided by van Manen’s methodology, employed all six lifeworld existentials: lived space, lived body, lived time, lived self-other, lived thing, and lived cyborg. Semi-structured interviews were conducted with 21 women diagnosed with psychosis at two hospitals in Riyadh, KSA. Data collection included 13 audio-recorded interviews and eight documented via field notes, supplemented by creative methods such as drawings, poems, and written texts analyzed using van Manen’s vocative method. All Arabic data were professionally translated and verified for accuracy. **Results**: Three overarching themes emerged. First, women’s lived experiences of first-episode psychosis highlighted the process of understanding causes and developing insight during onset. Second, experiences during admission and follow-up revealed the impact of clinical encounters, nursing care, and the critical need for therapeutic healing spaces. Third, living with psychosis in the community emphasized the complexities of medication adherence, family dynamics, and the pursuit of recovery through education, employment, and religious practice. **Conclusions**: The participants articulated user-based recovery perspectives, including empowerment, shared decision-making, and hope, which contrasted sharply with the service-based approaches they received. Culturally specific stressors and pervasive stigma shaped every phase of their journey. To the authors’ knowledge, no prior study has examined this population using a hermeneutic phenomenological framework; these findings provide a women-focused, culturally situated evidence base for developing gender-specific recovery models and enhanced discharge planning within the KSA mental health system.

## 1. Introduction

Psychosis represents a profound alteration in an individual’s relationship with reality, characterised by hallucinations, delusions, disorganised thinking, and disruptions to social functioning [1]. While the consumer experience of psychosis and recovery has received increasing attention in Western research [2,3,4], there remains a significant gap in understanding women’s lived experiences of psychosis within specific cultural contexts, particularly in the Kingdom of Saudi Arabia (KSA).

The KSA context introduces distinctive considerations that shape every dimension of the psychotic experience [5,6]. KSA’s culture follows an orthodox interpretation of Islam that significantly impacts individuals’ performance of their roles in the community [7]. The holy Quran and Hadith are used as the main sources of guidance in daily life and underpin all laws, knowledge, and spiritual experiences [8]. Individuals are viewed mainly as extensions of their immediate and extended families, and family cohesion is emphasised above individual interests [8]. For women, these cultural dynamics create specific challenges: the attribution of mental illness to supernatural causes such as jinn possession remains prevalent [9]; mental illness in women carries heightened stigma related to marriageability, family honour, and gender roles and women face greater barriers to seeking mental health treatment than men [10].

Studies of psychosis prevalence in KSA women are limited. The most recent cross-sectional study found that 45.5% of female inpatients and 20.6% of outpatients were diagnosed with primary psychotic disorders [11]. Duration of untreated psychosis in women was found to be longer than in men, suggesting that seeking treatment is delayed for women, possibly because a husband might believe it is better to keep his wife’s illness secret to preserve his standing in society [12]. Family responses range from rejection and divorce to concealment and denial [13,14]. Arab women face unyielding social constructions that impede access to mental health treatment [15].

Despite these distinctive influences, studies specifically exploring KSA women’s lived experiences of psychosis remain absent from the published literature. While parallel investigations have been conducted in other Muslim-majority contexts, including Pakistan, Iran, and Indonesia, where cultural and religious frameworks share certain similarities with the KSA, none has examined the intersection of Saudi gender norms, orthodox Islamic practice, and psychotic illness from the perspective of women themselves [16].

This study addresses the identified gap by exploring KSA women’s lived experiences across the full trajectory of psychosis, from first episode through hospitalisation to community living after discharge. To the authors’ knowledge, no prior published study has employed this women-focused, hermeneutic phenomenological approach within the specific sociocultural context of Saudi Arabia. The study aimed to explore the lived experiences of KSA women with psychosis across three phases: first-episode onset, admission to or follow-up from a mental health facility, and living in the community after discharge.

## 2. Materials and Methods

This study employed a hermeneutic phenomenological design guided by the methodology of van Manen [16,17,18]. The research was structured around all six lifeworld existentials to provide a comprehensive exploration of the participants’ experiences. These include the lived body (corporeality), which was central to understanding the women’s embodied experiences of psychotic symptoms, and the lived self-other (relationality), which revealed the impact of relationships with family, nurses, and the community. The lived time (temporality) existential illuminated the disruption of developmental timelines caused by the onset of psychosis, while lived space (spatiality) explored the influence of healthcare environments. Finally, lived thing (materiality) and lived cyborg (technology) examined the role of material events and technology in triggering or exacerbating symptoms.

To further interpret the women’s recovery experiences, the study integrated the recovery framework proposed by Schrank and Slade [19]. This framework distinguishes between service-based recovery, which focuses on clinical outcomes and medication compliance, and user-based recovery, which emphasizes empowerment, hope, and personal meaning. This dual perspective allowed for a nuanced interpretation of the care approaches the women encountered versus their own recovery aspirations.

The study was conducted at two hospitals in Riyadh, Saudi Arabia. Hospital A is a specialist mental health facility consisting of female short-stay and long-stay wards, while Hospital B is a general hospital with a single female psychiatric inpatient ward and a mental health outpatient department. A total of 21 Arabic-speaking women with a confirmed diagnosis of psychosis participated in the study, with 11 recruited from Hospital A and 10 from Hospital B. Participants were identified through lists provided by nursing professionals, and their clinical stability was assessed by nursing staff prior to recruitment to ensure they could safely engage in the research process.

Data were collected through semi-structured, in-depth interviews conducted in Arabic by the researchers, who were native Arabic speakers and qualified mental health nurses. The interview guide included open-ended questions covering each phase of participants’ experiences (onset, hospitalisation, and post-discharge community living), with follow-up probes used to explore emerging themes. Researchers remained flexible and followed participants’ narratives, allowing unanticipated themes to emerge. Thirteen interviews were audio-recorded, predominantly from Hospital B, while eight interviews from Hospital A were documented via detailed field notes due to institutional ethical and privacy restrictions on audio recording in inpatient wards. Audio recording was not offered as an alternative at Hospital A because institutional governance explicitly prohibited it across all ward-based research. In these cases, researchers wrote verbatim-style field notes during and immediately after each interview, capturing direct quotations, observed affect, and contextual observations. This approach is recognised as a methodologically acceptable alternative in qualitative research when recording is not possible [17,18]. To address the potential for reduced data richness, the analytical team cross-checked field notes with emerging themes through a reflexive audit process. Thematic coherence across field-note and recorded data sets was strong. The average interview duration was approximately 25 min; some sessions were shorter due to poverty of speech, a negative symptom of psychosis, or external time constraints.

As a Supplementary Materials collection strategy, women were invited, but not required, to produce creative materials about their experiences, including drawings, poems, and written texts. Of the 21 participants, six produced such materials (two drawings, two poems, and two written texts). These materials arose spontaneously during or after the interview, when participants independently expressed a desire to communicate through non-verbal or written means. Researchers provided paper and pens and explained that any form of creative expression was welcome, with no expectations regarding format or content. These creative materials were interpreted using van Manen’s lived thoroughness, nearness, intensification, pathic power, and epiphany, ensuring that the key words and images produced by the women were given their full phenomenological value and vivid presence.

The analysis followed van Manen’s [18] selective highlighting approach. Themes were identified, clustered, and reviewed by the research supervisors before being structurally analyzed through the lens of the six lifeworld existentials. This multi-layered process combined the descriptive highlighting of participant voices with the interpretive power of vocative analysis for the creative texts, ensuring a deep and rigorous engagement with the lived experience of psychosis.

The study was conducted in accordance with the ethical principles of the Declaration of Helsinki. Ethical approval was obtained from the Ministry of Health Research Committee for Hospital A and Hospital B (IRB Log Number: 17-127E). Written informed consent was obtained from all participants prior to data collection. When participants lacked decision-making capacity, written informed consent was obtained from their legally authorized guardians in accordance with the IRB-approved protocol. To ensure confidentiality, pseudonyms were assigned to participants, and all data were securely stored in password-protected files accessible only to the research team.

## 3. Results

### 3.1. Participants’ Demographic and Clinical Characteristics

This section outlines the demographic and clinical characteristics of the women diagnosed with psychosis who participated in the study. A total of 21 participants were recruited from two psychiatric hospitals (Hospital A and Hospital B), encompassing both inpatient units and outpatient departments (OPD). The inclusion of participants across these settings enabled the study to capture a broad range of clinical and lived experiences.

As presented in Table 1, participants ranged in age from 21 to 56 years, reflecting variation across different life stages. The sample was predominantly composed of Saudi nationals, with one participant from Egypt, providing limited but relevant cultural diversity. Educational attainment varied considerably, ranging from illiteracy to university-level education, while most participants were not engaged in formal employment at the time of the study. Marital status was similarly diverse, including single, married, divorced, and widowed women, with a number of participants reporting having children.

Clinical characteristics also demonstrated notable variation. The reported age of first episode ranged widely, suggesting differences in illness onset and potentially in illness trajectories. Additionally, participants were distributed across inpatient and outpatient settings, which may reflect differences in illness severity, access to care, and support systems.

Overall, the characteristics summarised in Table 1 highlight the heterogeneity of the sample. This diversity is analytically valuable, as it enables a more nuanced exploration of the lived experiences of women with psychosis within the Saudi context.

### 3.2. Qualitative Themes of Women’s Experiences of Living with Psychosis

This section presents the key themes and sub-themes that emerged from the qualitative analysis of women’s experiences of living with psychosis in the Kingdom of Saudi Arabia (KSA). Using an inductive thematic approach, the analysis identified three overarching themes that reflect the temporal and experiential trajectory of psychosis, spanning from initial onset to post-discharge life in the community.

As illustrated in Table 2, the first theme captures women’s lived experiences of first-episode psychosis, including their interpretations of causation and their evolving awareness of symptoms. The second theme focuses on participants’ experiences within mental health services, encompassing admission or follow-up care, interactions with healthcare providers, and perceptions of the therapeutic environment. The third theme reflects women’s experiences following discharge, highlighting ongoing challenges and recovery processes within the community context.

Each theme is further elaborated through interconnected sub-themes that provide deeper insight into participants’ meaning-making processes, coping strategies, and interactions with social and institutional structures. Together, the themes presented in Table 2 offer a comprehensive framework for understanding the multifaceted and evolving nature of living with psychosis among women in the Saudi context.

### 3.3. Theme 1: Women’s Lived Experiences of First-Episode Psychosis

#### 3.3.1. Sub Theme 1: Understanding the Cause of Psychosis

The women’s views revealed their understanding of what caused their psychosis. Many cited continuous or exceptional stress as the trigger. Psychosis onset predominantly occurred during adolescence and young adulthood, a critical developmental period for identity, independence, intimate relationships, study, and career plans [20]. Zahra suggested her struggle to cope during university contributed to onset. Abeer described psychosis beginning at school. These experiences align with McFarlane et al.’s [21] finding that psychosis is generally induced in biologically vulnerable individuals by significant life stresses, including role transitions, social isolation, family conflict, and stigma.

Nadia described the beginning of her experience with hallucinations, noting her family’s lack of support: she could not speak about hallucinating because her family did not understand. This concealment reflects McGrath et al.’s [22] finding that once some women understand they have a mental health problem, they tend to conceal it due to fear of hospitalisation, loss of custody, and feelings of guilt and shame.

#### 3.3.2. Experiencing Onset with Developing Insight

As symptoms emerged, some women experienced the impact of their relationships with others, particularly family, in the development of symptoms. The women’s understanding of and insight into the onset varied. Some developed awareness that their experiences were symptoms of illness, while others attributed them to supernatural causes, consistent with cultural beliefs about jinn possession. The coexistence of clinical and culturally grounded explanatory models within individual narratives was a distinctive finding.

### 3.4. Theme 2: Experiences After Admission or Follow-Up

#### 3.4.1. Sub Theme 1: Admission or Follow-Up Experiences

The transition to hospitalisation was experienced as disorienting and sometimes frightening. Joud described her first admission:

The first time I was admitted to the hospital; I saw a consumer holding a knife to cut an orange. She looked strangely at me, [and] I thought she was a criminal. Other consumers [tried to] get to my room and speak with me while I did not know them; it was terrifying. I felt that they were a family and that I was an intruder.

(p. 3)

This account reveals the visceral fear of entering an unfamiliar environment where consumers of different acuity levels are co-located. Karima similarly expressed: They did not separate…stable cases and other early-stage cases, which was the most annoying thing…. The absence of high-dependency and low-dependency areas in Hospital B meant that newly admitted women were exposed to acutely unwell consumers, a situation the women found distressing and counterproductive to recovery.

#### 3.4.2. Sub Theme 2: Experiencing Symptoms After Admission

Women described ongoing psychotic symptoms during hospitalisation. One woman reported: ‘At my room in the hospital, I hear the TV sounds coming from the hall, but I hear different words…I used to hear sounds and explain it in another way.’ This account reveals how environmental stimuli within the hospital triggered altered perceptions, with television sounds being reinterpreted through the prism of psychosis, highlighting the importance of sensory environment management within psychiatric facilities.

CCTV in women’s rooms and the replacement of physical observation with electronic devices were identified as tending to escalate specific psychotic symptoms. This finding reveals how technology designed for safety purposes may paradoxically worsen the condition it is meant to monitor.

#### 3.4.3. Sub Theme 3: Experiences with Services and Nursing Care

Women offered diverse perspectives on nursing care. Hamida from Hospital A indicated that nurses were good generally, but they sometimes ‘abused her by shouting at her, pushing, hitting her, and asking her to go away.’ Hala from Hospital B described her experience with seclusion: ‘When I was admitted here, there was a nurse who detained us in the seclusion room, which made me get worse’ (p. 1). Najat framed restraining as a form of punishment. These accounts of seclusion and restraint as harmful and punitive are consistent with evidence that adverse experiences of coercive measures can cause enduring negative attitudes toward mental health care and promote trauma [23,24].

Najat felt that the staff ‘repressed her and were not flexible’ and that the ‘system of care at the hospital did not help her recover.’ Unprompted during her interview, Najat produced several pages of handwritten Arabic notes entitled ‘How the Nurses Should Deal with the Consumers’. These notes were structured as a practical guide with numbered points, covering: respectful communication with consumers; the importance of explaining diagnoses and medications in accessible language; the need for peer support groups on the ward; and the role of consumer involvement in co-designing treatment plans. She wrote: ‘The consumer should [take] the strongest and most cohesive part to put the treatment process into its best position. Lectures and seminars for the consumers are needed to increase consumer’s awareness and reinforce their physical and mental health.’ She also noted that nurses should be trained in active listening and should never dismiss a consumer’s account of her experience as mere symptom. The spontaneous production of this document, composed during hospitalisation without any researcher prompting, suggests that Najat had developed a sophisticated understanding of recovery-oriented care through her own experiential knowledge, despite never having encountered the formal literature. This act of consumer advocacy from within the hospital constitutes an independent, empirically grounded articulation of user-based recovery principles.

Zahra discussed how nurses could address consumer needs by offering ‘advocacy and support based on mental healthcare policy to decrease consumer’s misunderstandings.’ The language barrier emerged as a significant concern: women in Hospital B expressed discontent with nurses being unable to understand their language, and they wished for more Arabic-speaking staff. Zahra powerfully stated: ‘The language is a barrier between us, and there was no intimacy or…home girl feelings, [nurses are strangers]’ (p. 13).

Alya’s Poem (Vocative Analysis), Alya, from Hospital A, wrote a poem during her interview:

You know my state, God. I ask you to give me what I wish. Help me to be happy.

Alya’s poem highlights her sadness and accumulated stressors, emphasising her wish to improve and finish her treatment. Her hope to recover and spirituality towards God are main components of user-based recovery [19]. While she did not write directly about the ward’s services or staff, her tone suggests she was not happy in the hospital, indicating her level of satisfaction with services and care.

#### 3.4.4. Sub Theme 4: The Need for a Healing Space

The need for healing spaces within mental health facilities was stressed by many women, converging with nurses’ perspectives. Women called for gardens, green spaces, fresh air, and homelike atmospheres. One consumer stated: ‘I wish if they could provide gardens and liberate us to get out to the garden of the hospitals.’ Joud recommended separating consumers by acuity level. These consumer perspectives provide powerful evidence for facility redesign, and women’s involvement in planning their own spaces was recommended.

### 3.5. Theme 3: Experiences of Living with Psychosis After Discharge

#### 3.5.1. Sub Theme 1: Medication Adherence

The development of understanding of the importance of medication adherence varied among women. The impact of relationships, including with staff and family, on medication behaviour was significant. Zahra described how her father, educated about medication by her psychiatrist, was then able to help her regarding side effects. This single positive example of family psychoeducation reinforcing medication adherence underscores the potential of such interventions. Other women had limited awareness of the importance of adherence, which may have contributed to their belief that they did not need medication.

#### 3.5.2. Sub Theme 2: Living with Family, Friends, and the Community

Post-discharge life was profoundly shaped by family responses and community stigma. While women did not use the term ‘stigma,’ their views reflected its impact: experiences of being rejected, divorced, or abused by others, along with associated social problems and deterioration of relationships, highlighted the social stigma experienced by women which may hinder recovery from psychosis.

Hamida reported: ‘My daughters told me “You are crazy; you are like a child”’, while her husband told her ‘You are okay, you do not have an illness’ when she asked to be taken to hospital. This dual response, mockery and denial, illustrates how stigma operates within family systems to simultaneously devalue the person and deny the reality of illness.

Family knowledge potentially reinforcing recovery, particularly concerning supporting medication adherence, was noted. However, most women suggested that their families lacked knowledge about dealing with them after discharge, which hindered their recovery. Family psychoeducation can address problems of medication non-adherence, misunderstanding of psychosis, and may also reduce family stigma.

#### 3.5.3. Sub Theme 3: Recovery from Symptoms and Developing Insight

Women at different stages showed different levels of insight. Some described developing understanding of their illness over time. The sub-theme of insight into psychosis appeared across all three major themes, reflecting a developmental trajectory of increasing self-awareness. Women believed in elements of user-based recovery: adopting positive attitudes, having belief in themselves, accepting psychosis as a manageable illness, and committing to long-term medication.

#### 3.5.4. Sub Theme 4: Recovery Through Education, Employment, and Religion

Recovery experiences after discharge included the importance of education, employment, and religion. Afaf stated: ‘Now I am recovered and become normal, I will continue my education for my future career’ (p. 1). Najat believed that improving herself through employment would accelerate recovery. Abel et al. [25] reported that women with psychosis are more likely to be employed than men and more likely to retain social support systems, which may explain the confident tone taken by these women.

Religion, spirituality, and faith in God were viewed as enablers of recovery. In KSA’s Islamic community, religion influences most aspects of life, including responses to illness. Saged et al. [26] found that 98% of Muslim individuals with mental illness believed that deep faith in Allah and reading or listening to the Quran aided recovery. While this finding may not surprise in a KSA context, it underscores the importance of incorporating spiritual dimensions into recovery-oriented care.

Jumana’s Drawing: ‘Broken Heart’ (Vocative Analysis). Jumana from Hospital B drew a picture she titled ‘Broken Heart’. The drawing depicted a single large heart, rendered in red crayon, split down the centre by a jagged black line. On either side of the fracture, smaller marks extended outward like radiating cracks. The background was left white and empty. Through vocative analysis, the compositional elements carried strong phenomenological weight. The choice of red, saturated and dominant, communicated the intensity of affect. The jagged break conveyed violence and rupture rather than resolution. The empty surrounding space evoked isolation and an absence of relational anchoring. The radiating cracks suggested the fracture was spreading rather than contained. Vocative analysis revealed five dimensions: lived thoroughness (the total occupation of the emotional field by pain), nearness (the immediate, pressing quality of heartbreak as lived right now), intensification (the escalating cracks as a visual grammar of worsening distress), pathic power (the drawing’s capacity to move the viewer into empathic resonance), and epiphany (the recognition that this woman’s experience exceeds what verbal language could fully hold). The drawing served as a non-verbal articulation of experiences that words alone could not fully capture, reinforcing the sub-theme of emotional rupture within family and community relationships following discharge.

## 4. Discussion

### 4.1. First Women-Focused Study in KSA

This study explored KSA women’s lived experiences of psychosis and provides culturally situated insights into the trajectory from onset through hospitalisation to community living. To the authors’ knowledge, no prior published study has investigated this specific population using a hermeneutic phenomenological framework. The findings reveal that while some patterns, including the role of stress in triggering psychosis, the impact of stigma on recovery, and the centrality of medication adherence, are consistent with international literature, the KSA context introduces dimensions not previously documented for this population.

### 4.2. Service-Based Versus User-Based Recovery

A critical finding is the disconnect between the care approaches received by women and their own recovery aspirations. The nurses’ care was characterised as ‘physical and custodial care only,’ not empowering women to be independent or develop self-help [19]. The nurses’ current practices ‘tend to not empower women to be independent and develop self-help and control, nor to engage or educate women and their families,’ suggesting care through the lens of service-based recovery only.

Yet the women themselves articulated user-based recovery perspectives [27,28,29]. Najat advocated for consumer involvement in treatment planning. Women described hope, faith, and desire to return to education and employment. Alya’s poem expressed spirituality as a recovery resource. These consumer perspectives align with international evidence that user-based recovery from psychosis is both unique to each individual and a continuous experience rather than a final endpoint [2].

This gap between service-based care delivery and user-based recovery aspirations constitutes a fundamental challenge for KSA mental health services. Addressing this gap requires system-level transformation, developing a model of recovery suitable for the KSA context which incorporates religious and cultural dimensions while embracing the empowerment principles central to user-based recovery [30,31,32].

### 4.3. Gender-Specific Dimensions

The gendered dimensions of psychosis in KSA are profound. Women’s delayed help-seeking, driven by family concealment to preserve male honour, extends the duration of untreated psychosis, with consequences for long-term outcomes. The intersection of mental illness stigma with gender norms creates a ‘double burden’: women are not only stigmatised for having a mental illness but face additional consequences related to marriage prospects, custody, and family standing [33,34]. The finding that some women’s delusions reflected genuine concerns, about polygamy, divorce, and loss of children, rather than purely pathological ideation [14] challenges simplistic clinical interpretations. It should be noted that certain stigma experiences described by participants are not exclusively gendered. Fear of hospitalisation, illness concealment, and the social consequences of a psychiatric diagnosis are documented across genders in the Arab world and in collectivist societies more broadly [33,34]. What is specifically gendered in this study’s findings is not the existence of stigma, but its particular content and consequences for women: marriageability, polygamy, custody, and male gatekeeping of help-seeking. These findings therefore complement rather than contradict the broader stigma literature, while adding a culturally specific gendered layer not previously documented for the KSA population.

### 4.4. The Role of Family

Family emerged as both the most significant potential resource and the most significant potential barrier to recovery [35,36,37]. Families who were educated and engaged supported medication adherence and community reintegration. Families who lacked understanding perpetuated stigma, delayed treatment, and impeded recovery [34,38]. This duality supports the recommendation for comprehensive family psychoeducation programmers that are culturally tailored to the KSA context, addressing not only clinical knowledge but also the cultural and religious beliefs that shape family responses.

### 4.5. Implications for Practice: Gender-Specific Recovery Models

To address the unique needs of women with psychosis in Saudi Arabia, clinical practice should transition toward a gender-sensitive recovery model that integrates Islamic spirituality and active family engagement. Central to this shift is the promotion of consumer involvement in treatment planning and the implementation of acuity-based ward allocation to reduce patient distress during admission. Furthermore, there is an urgent need to minimize the reliance on seclusion and restraint, which participants in this study perceived as punitive rather than therapeutic. Finally, therapeutic communication must be prioritized by ensuring Arabic language proficiency for all nursing staff and establishing structured discharge planning that includes family psychoeducation and consistent community follow-up. Family psychoeducation programmes should be delivered by trained mental health nurses and community psychiatric workers as structured multi-session interventions beginning prior to discharge. Content should address: the biological and psychosocial aetiology of psychosis (to counter jinn-based explanatory models); medication adherence; recognition of relapse signs; and constructive communication strategies. Engaging resistant families requires cultural respect rather than confrontation, drawing on Islamic principles of compassion and family duty (amanah). Where family members are themselves sources of abuse, coercion, or denial, the clinical team must establish independent advocacy and safety planning, and identify alternative support networks including peer support groups and community welfare services under Saudi Vision 2030.

### 4.6. Implications for Policy

Under the strategic framework of Saudi Vision 2030 [39,40], these findings provide an empirical basis for systemic policy reforms within the Kingdom’s mental health sector [40]. A primary policy priority should be the development of a national recovery model that mandates family psychoeducation as a standard component of discharge planning. To foster a truly recovery-oriented system, national health authorities should establish formal mechanisms for consumer participation in service design and evaluation, alongside the creation of independent advocacy services for mental health consumers.

Furthermore, public health policies must prioritize community education campaigns specifically designed to reduce gendered stigma and promote the early identification of individuals vulnerable to psychosis. By institutionalizing these reforms, the Ministry of Health can ensure that mental health services in Saudi Arabia move beyond clinical stabilization toward a holistic, rights-based approach that empowers women and their families throughout the recovery journey.

### 4.7. Implications for Nursing Education

To align nursing practice with the lived experiences of women, curricula should prioritize the inclusion of consumer voices and user-based recovery perspectives. This requires a shift toward gender-sensitive mental health content that accurately reflects the unique cultural and religious context of Saudi Arabia. Furthermore, nursing education must provide intensive training in therapeutic communication and de-escalation techniques, emphasizing the development of alternatives to restrictive practices. Finally, a robust focus on cultural competency is essential to help students understand the complex intersection of faith, family dynamics, and mental illness. By integrating these elements, nursing education can prepare a workforce that is not only clinically proficient but also culturally humble and recovery-oriented.

### 4.8. Limitations

Several limitations warrant consideration when interpreting these findings. First, the use of field notes rather than audio recordings for Hospital A participants, due to institutional restrictions, means data from these eight women may lack the precise verbatim quality of transcribed interviews. Although researchers wrote contemporaneous, verbatim-style notes, some loss of conversational nuance is unavoidable. Future studies should advocate for revised institutional recording policies. Second, as with all hermeneutic phenomenological research, findings are not intended to be statistically generalisable [18]; rather, they offer transferable theoretical insights applicable to similar sociocultural contexts. Third, recruitment was limited to two hospitals in Riyadh, which may not reflect the full diversity of KSA mental health service provision. Fourth, some interviews were brief due to poverty of speech, a negative symptom of psychosis, or external time constraints; future studies should explore flexible scheduling. Fifth, only six of 21 participants produced creative materials, limiting the representativeness of the vocative data; however, these accounts provided distinctive phenomenological depth that enriched the overall analysis. Finally, participants may have moderated negative accounts of care quality in the presence of researchers known to nursing staff at the same hospitals. Member checking and reflexive bracketing were employed to mitigate this risk.

## 5. Conclusions

This study explored KSA women’s lived experiences of psychosis across three phases: first-episode onset, hospitalisation or follow-up care, and community living after discharge. The findings make four substantive contributions. First, they establish that women’s psychosis in KSA is shaped by a distinctive constellation of culturally specific stressors, including supernatural explanatory models, gendered stigma tied to marriageability and family honour, and male gatekeeping of help-seeking, that collectively extend the duration of untreated psychosis. Second, the study reveals a fundamental disconnect between the service-based, custodial care women received and their own user-based recovery aspirations centred on empowerment, shared decision-making, spiritual resources, education, and employment. Third, the findings identify family as the most consequential dual variable in recovery: educated and engaged families accelerated recovery, while families characterised by stigma, denial, or abuse impeded it. Targeted family psychoeducation, co-designed with cultural and religious sensitivity, is the most actionable short-term intervention available. Fourth, the study demonstrates the value of van Manen’s hermeneutic phenomenology, including vocative analysis of creative materials, for generating culturally situated data in populations where verbal articulation may be constrained by stigma or symptom severity. These findings provide an empirical basis for developing gender-specific recovery models, reforming nursing education to incorporate consumer perspectives and cultural competency, and informing mental health policy under Saudi Vision 2030. Research priorities include multi-site KSA studies, longitudinal follow-up across recovery phases, and co-design of culturally adapted recovery-oriented interventions with women who have lived experience of psychosis.

## Figures and Tables

**Table 1:** Women with Psychosis Biographies.

No.	ID	Pseudonym	Age	Hospital	MH Unit	Marital Status	Children	Education Level	Job	First Episode (Age)	Nationality
1	WWP1	Safiya	29	B	Inpatient	Married	Y	Intermediate	N	17	Saudi
2	WWP2	Zahra	40	B	OPD	Married	Y	Secondary	N	17	Saudi
3	WWP3	Jana	42	B	Inpatient	Single	N	Secondary	Y	20	Saudi
4	WWP4	Hala	50	B	OPD	Single	N	University	N	25	Saudi
5	WWP5	Joud	21	B	OPD	Single	N	Secondary	N	17	Saudi
6	WWP6	Lama	37	B	OPD	Divorced	Y	Secondary	N	20	Saudi
7	WWP7	Nadia	33	B	OPD	Single	N	Primary	N	18	Saudi
8	WWP8	Abeer	27	B	OPD	Single	N	University	N	20	Saudi
9	WWP9	Karima	40	B	OPD	Married	Y	Secondary	N	17	Saudi
10	WWP10	Maha	40	B	OPD	Married	N	Primary	N	15	Saudi
11	WWP11	Sahar	47	B	OPD	Married	Y	Secondary	N	41	Saudi
12	WWP12	Afaf	26	B	OPD	Single	N	Secondary	N	16	Saudi
13	WWP13	Nahla	25	B	OPD	Single	N	Intermediate	N	17	Saudi
14	WWP14	Hamida	30	A	Inpatient	Divorced	Y	Illiterate	N	20	Saudi
15	WWP15	Alya	50	A	Inpatient	Married	Y	Intermediate	N	15	Saudi
16	WWP16	Halima	48	A	Inpatient	Divorced	Y	Secondary	N	26	Saudi
17	WWP17	Eliana	52	A	Inpatient	Divorced	Y	University	N	25	Egyptian
18	WWP18	Jumana	42	A	Inpatient	Married	Y	Intermediate	N	13	Saudi
19	WWP19	Dalia	56	A	Inpatient	Divorced	Y	Primary	N	20	Saudi
20	WWP20	Salma	35	B	OPD	Widowed	Y	University	Y	24	Saudi
21	WWP21	Najat	31	A	Inpatient	Single	N	University	—	27	Saudi

**Table 2:** Themes representing KSA women’s experiences of living with psychosis.

Theme	Sub-Themes
1. Women’s lived experiences of first-episode psychosis	1a. Understanding the cause of psychosis
	1b. Experiencing onset of symptoms with developing insight
2. Experiences after admission to or follow-up from a mental health facility	2a. Admission or follow-up experiences
	2b. Experiencing symptoms and insight after admission
	2c. Experiences with services and nursing care
	2d. The need for a healing space
3. Experiences of living with psychosis in the community after discharge	3a. Medication adherence
	3b. Living with family, friends, and the community
	3c. Recovery from symptoms and developing insight
	3d. Recovery through education, employment, and religion

## Data Availability

The datasets generated and/or analyzed during the current study are not publicly available due to participant confidentiality and ethical restrictions but are available from the corresponding author on reasonable request.

## Supplementary Materials

The following supporting information can be downloaded at: https://www.mdpi.com/article/10.3390/healthcare14131970/s1.

## Abbreviation

The following abbreviation is used in this manuscript:

KSA	Kingdom of Saudi Arabia
