# Supplementary material for: Voices from Within: Saudi Arabian Women’s Lived Experiences of First-Episode Psychosis, Hospitalisation, and Recovery Pathways
Source: Healthcare (Basel). 2026 Jul 2;14(13):1970. doi: 10.3390/healthcare14131970 (PMC13361423; doi:10.3390/healthcare14131970)
Supplement: Supplementary file 1 [file healthcare-14-01970-s001.zip › healthcare-4257426-supplementary.pdf]

SUPPLEMENTARY MATERIALS

SUPPLEMENTARY FILE B: INTERVIEW GUIDES

B.1 — Interview Guide for Nurses

Demographics (collected prior to interview):

- Age
- Level of nursing education (Diploma / Higher Diploma / Bachelor's)
- Specialty of awarded degree (General Nursing / Mental Health Nursing)
- Years of experience in mental health nursing
- Mental health training received (Yes/No)
- If Yes: Focus of training (Principles of mental health care / Symptomatology and mental disorders / Psycho-social interventions / Psychotropic medications)

Interview Questions:

1. Can you tell me about your experience of working as a nurse in this mental health facility?
2. What do you understand about psychosis? How would you describe it?
3. Can you describe the experience of caring for women with psychosis in this facility?
4. How would you describe the physical environment of the ward/OPD where you work? How does it affect your care provision?
5. What is it like to communicate with women who have psychosis? What challenges do you face?
6. Can you tell me about your experiences of communicating with consumers' families?
7. How do you feel emotionally when caring for women with psychosis? Can you describe a time when providing care was particularly emotionally challenging?
8. Have you experienced any situations involving transference or countertransference with consumers? Can you describe what happened?
9. What do you know about medication adherence in women with psychosis? What factors do you think affect it?
10. Is there anything else you would like to share about your experience of caring for women with psychosis?
11. What improvements would you suggest for the mental health services here?

Probes used throughout: "Can you tell me more about that?"; "How did that make you feel?"; "Can you give me an example?"; "What do you mean by…?"

B.2 — Interview Guide for Women with Psychosis

Demographics (collected prior to or after interview):

- Age range
- Marital status
- Diagnosis
- Number of admissions
- Setting (inpatient / OPD)

Interview Questions:

1. Can you tell me about your experience of your illness?
2. When did you first start to feel unwell? What was that like?
3. What do you think caused your illness?
4. Can you tell me about your experience of being admitted to this hospital / coming to this clinic?
5. How would you describe the nursing care you receive here?
6. How do you feel about the environment/space of this ward or clinic?
7. Can you tell me about your experience with medication?
8. How has your illness affected your life at home and in your community?
9. What has helped you in your recovery? What has made it harder?
10. Is there anything you would like to change about the care you receive?
11. Is there anything else you would like to share about your experience?

Supplementary creative methods (offered during interviews):

- "Would you like to draw a picture about your experience?"
- "Would you like to write something about your thoughts or feelings?"

SUPPLEMENTARY FILE C: PARTICIPANT DEMOGRAPHICS — NURSES ($N = 21$)

Table C.1: Demographic Data of Nurse Participants

Item	Number
**Total number of nurses**	21
Nurses from Hospital A	11
Nurses from Hospital B	10
**Average age**	36.9 years
**Level of nursing education**	
Diploma (2–3 years post-secondary)	13
Bachelor's degree (4 years post-secondary)	8
**Specialty of awarded degree**	
General Nursing	20
Mental Health Nursing	1
**Average years of experience in mental health nursing**	9.7 years
**Mental health training received**	
Yes	19
No	2
**Focus of training (for the 19 nurses who received training)**	
Principles of mental health care	18
Symptomatology and mental disorders	17
Psycho-social interventions	12
Psychotropic medications	18

Table C.2: Nurse Pseudonyms by Hospital and Interview Language

Pseudonym	Hospital	Interview Language	Ward Assignment
Madeeha	A	Arabic	Inpatient
Fadwah	A	Arabic	Inpatient
Sabah	A	Arabic	Inpatient
Sarah	A	Arabic	Inpatient
Manahel	A	Arabic	Inpatient
Ahd	A	Arabic	Inpatient
Amani	A	Arabic	Inpatient
Fawziyah	A	Arabic	Inpatient
Haifa	A	Arabic	Inpatient
Mona	A	Arabic	OPD
Rawan	A	Arabic	OPD
Sophia	B	English	Inpatient
Catherine	B	English	Inpatient
Anna	B	English	OPD
Caroline	B	English	OPD
Grace	B	English	Inpatient
Maria	B	English	Inpatient

Helen	B	English	Inpatient
Jane	B	English	Inpatient
Olivia	B	English	OPD
Ruth	B	English	OPD

SUPPLEMENTARY FILE D: PARTICIPANT DEMOGRAPHICS — WOMEN WITH PSYCHOSIS (*N* = 21)

Table D.1: Women with Psychosis — Biographies

Pseudonym	Hospital	Setting	Data Collection Method	Key Features
WWP1–WWP10	A	Inpatient (short-stay or long-stay)	Field notes + drawings/writing	Rich accounts of repeated hospitalisations; some women produced drawings and written texts used in vocative analysis
WWP11	A	Inpatient	Audio-recorded	Single audio-recorded interview from Hospital A
WWP12–WWP21	B	Inpatient/OPD	Audio-recorded	Provided detailed audio-recorded narratives

Key participants referenced in manuscripts by pseudonym:

Pseudonym	Hospital	Key Contributions
Joud	B	Recommended separating consumers by acuity; described terrifying first admission
Zahra	B	Described language barriers with expatriate nurses; discussed onset at university
Abeer	B	Described psychosis onset at school
Nadia	B	Described family lack of support during hallucination onset
Karima	B	Discussed distress at co-location with acutely unwell consumers
Dalia	A	Produced written text about her thoughts
Alya	A	Wrote a poem about her distress and hope for recovery
Hamida	A	Drew a picture of a car; reported nurses' abusive behaviour
Najat	A	Produced notes on 'How the Nurses Should Deal with the Consumers'; advocated for consumer involvement in treatment
Jumana	B	Drew 'Broken Heart' expressing her emotional distress

SUPPLEMENTARY FILE E: INCLUSION AND EXCLUSION CRITERIA

Table E.1: Inclusion and Exclusion Criteria for Women with Psychosis

Criteria	Inclusion	Exclusion
Diagnosis	Clinical diagnosis of psychosis (confirmed by nursing staff using diagnostic records)	No confirmed diagnosis of psychosis
Setting	Admitted to or receiving follow-up at female inpatient wards or mental health OPDs at Hospital A or Hospital B	Not currently receiving care at study sites
Consent	Able to provide informed consent (or consent provided by guardian)	Unable to provide informed consent and no guardian available
Clinical status	Sufficiently stable to participate in an interview (as determined by nursing staff)	Acutely unwell to the extent that participation would cause distress or be clinically inappropriate
Language	Arabic-speaking	Non-Arabic-speaking

Table E.2: Inclusion and Exclusion Criteria for Nurses

Criteria	Inclusion	Exclusion
Role	Registered nurse currently providing direct nursing care to women with psychosis	Not currently providing direct care to women with psychosis
Setting	Working at female inpatient wards or mental health OPDs at Hospital A or Hospital B	Not working at study sites
Consent	Willing to provide informed consent	Unwilling to consent
Gender	Female (required by gender-segregated ward structure)	Male (males do not work on female wards in KSA)
